# Case Report: Kounis syndrome due to cryptopteran bite

**DOI:** 10.3389/fcvm.2024.1339514

**Published:** 2024-02-06

**Authors:** Rui Liao, Shengjie Cheng, Nan Xu

**Affiliations:** Department of Cardiology, The First People’s Hospital of Neijiang, Neijiang, China

**Keywords:** kounis syndrome, cryptopteran, case report, acute coronary syndrome (ACS), allergic reaction

## Abstract

**Background:**

Kounis syndrome is an acute coronary syndrome (ACS) caused by allergic reactions, including coronary artery spasm (type I) caused by allergies without coronary predisposing factors, pre-existing coronary atherosclerosis, and coronary artery disease. Anaphylaxis leads to plaque rupture or erosion leading to acute myocardial infarction (type II) and acute coronary stent thrombosis (type III). Here we share a case of Kounis syndrome type I caused by an allergy caused by a Cryptopteran bite.

**Case presentation:**

A 47-year-old woman was admitted to the hospital due to an insect bite for 2 days and chest distress for more than 3 h. Outside the hospital, electrocardiogram(ECG) showed sinus rhythm, ST-segment elevation in leads V1–V3, high-sensitivity troponin 2.54 ng/ml(0–0.5 ng/ml). One hour later, the ECG of the patient showed that the ST segment elevation of lead V1–V4 was 0.10–0.20 mV. Emergency coronary angiography showed coronary spasm and moderate lumen stenosis in the middle segment of left anterior descending artery (LAD). After treatment, the patient's symptoms were relieved, and the ST segment of lead V1–V4 of electrocardiogram returned to normal.

**Conclusion:**

Kunis syndrome is a life-threatening condition that can also cause myocardial ischemic injury in patients with or without coronary artery disease. Timely identification and anti-allergic treatment can achieve a good prognosis.

## Introduction

Kounis syndrome (KS) was described in 1991 by Kounis and Zavras (1). Kounis syndrome is an acute coronary syndrome (ACS) induced by the activation of mast cells and platelets under the condition of allergy, hypersensitivity, allergy or anaphylactoid reaction (2). At present, KS is divided into three types. Type I occurs when the coronary arteries are normal or nearly normal and the acute release of inflammatory mediators results in coronary spasm. Myocardial enzymes may be normal or progress to acute myocardial infarction (AMI). Type II refers to patients with atherosclerotic disease, in which acute release of inflammatory mediators can induce coronary artery spasm or with plaque erosion or rupture to manifest as AMI (2, 3). Type III includes patients with intracoronary stent thrombosis. Kounis syndrome is not rare, but most information comes from clinical case reports because the disease is underrecognized.

The wandering beetle belongs to the Coleopteran grape family. The family contains 3,847 genera, including Paederus, with more than 622 known species (4). Numbers increase significantly during the rainy season in July and August. Cryptopterans are usually large, moderately raised, and usually have two colors, prominent in black or blue and red, but sometimes the entire body is monochromatic in black, blue, or red. Cryptopteran has secretory glands that produce chemical secretions for defense, and its secretions are the main cause of cryptopteran dermatitis (5).

## Case presentation

A 47-year-old woman was admitted to the hospital with a 2-day bug bite and chest tightness for more than 3 h. Two days ago, the patient was accidentally bitten by Cryptopteran, and felt itchy skin. Then, she went to the local clinic for treatment. The itching symptom was relieved after treatment with related drugs (the specific drugs were unknown), but she felt skin pain and did not continue treatment. More than 3 h before admission, the patient felt pressuring sensation in the precordial area, slight shortness of breath, and no chest pain or discomfort. Normal body, measured blood pressure increased for more than five months. The highest systolic blood pressure was around 150 mmHg, and 30 mg nifedipine sustained-release tablets were taken intermittently. She denied any other medical history.

The body temperature was 36.3°C, respiration 20 times/min, pulse 100 times/min, blood pressure 144/88 mmHg. The oxygen saturation was 98%. There were patchy brown-red rashes on the neck. Physical examination of the heart, lung, abdomen, and nervous system showed no obvious positive signs.

**Figure 1:**
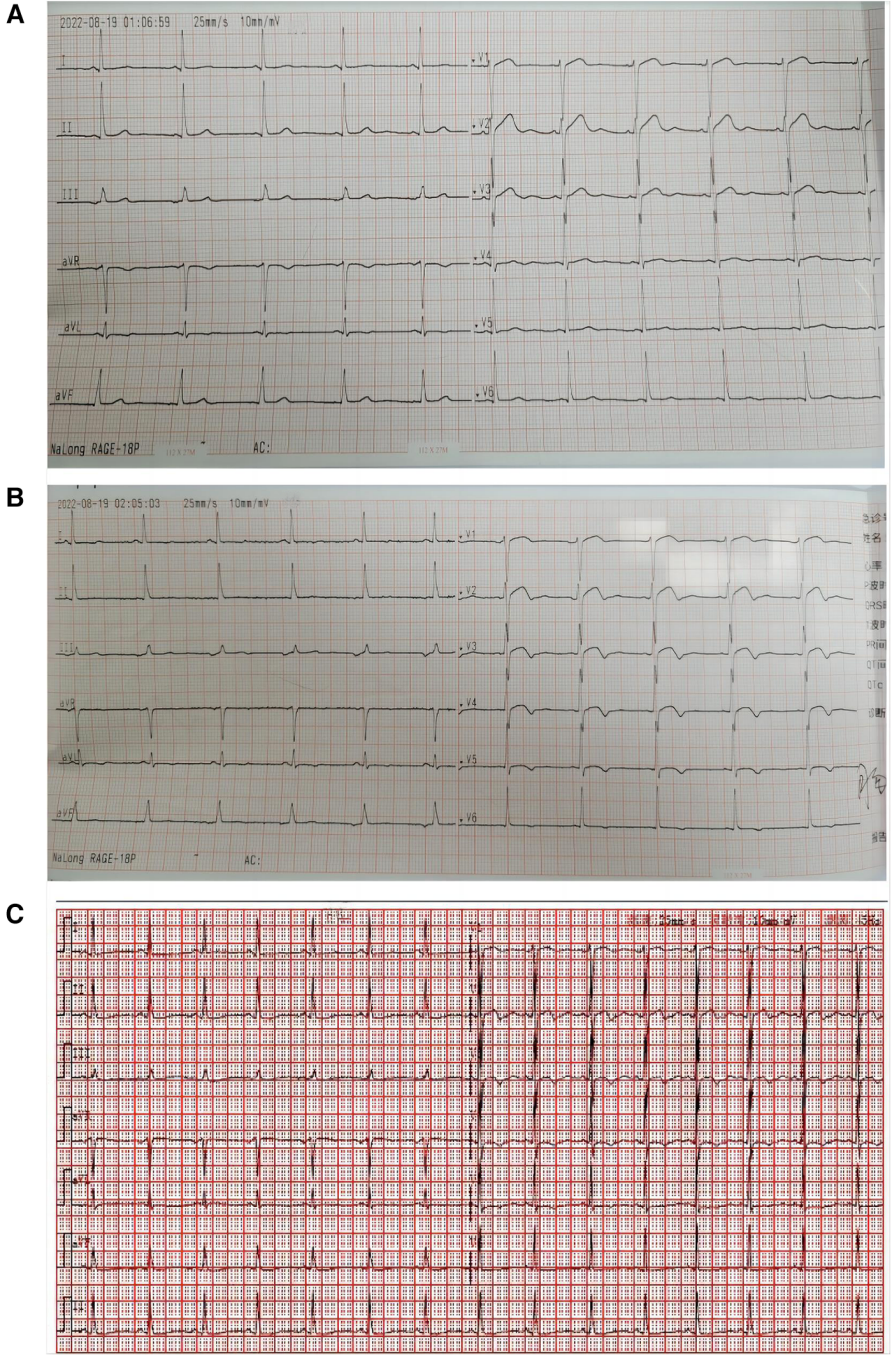
First electrocardiogram outside the hospital. (**A**) Fist electrocardiogram showed sinus rhythm and ST segment elevation in lead V1–V3. (**B**) An hour later, a repeat electrocardiogram showed sinus rhythm with ST-segment elevation in leads V1–V4. (**C**) Electrocardiogram of the patient after antiallergic treatment.

**Figure 2:**
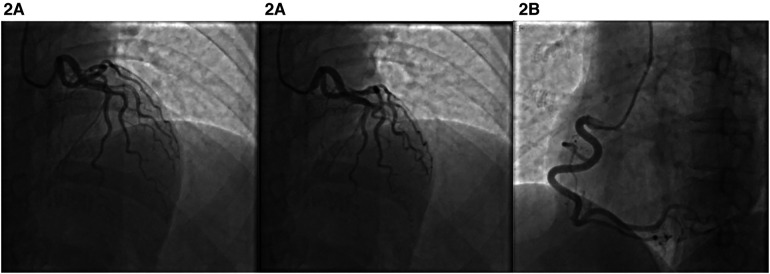
Emergency coronary angiography. (**A**) Muscle bridge and spasm in the middle segment of the LAD. (**B**) Right coronary artery.

Auxiliary examination: ECG showed sinus rhythm and ST segment elevation in lead V1–V3 (Figure 1A). An hour later, a repeat ECG showed sinus rhythm with ST-segment elevation in leads V1–V4 (Figure 1B). High-sensitivity cardiac troponin 2.54 ng/ml(0–0.5 ng/ml). The patient’s white blood cell count was 7.1 × 10^9^ (3.5–9.9 × 10^9^), the percentage of neutrophils was 57% (40.0%–75.0%), the eosinophil count was 0.67 × 10^9^(0.02–0.52 × 10^9^), the high-sensitivity C-reactive protein was 46.7 mg/L(0–10 mg/L), and the procalcitonin was 0.11 ng/ml(<0.05 ng/ml). Transthoracic echocardiography revealed a left ventricular ejection fraction of 67%, left atrium of 29 mm, left ventricle of 41 mm, interventricular septum of 11 mm, and right atrium of 36 × 28 mm.Emergency coronary angiography showed no left main coronary artery stenosis, myocardial bridge in the middle segment of LAD, moderate systolic lumen compression, TIMI3 blood flow, left circumflex artery(LCX) smooth patency, no plaque grade stenosis, TIMI3 blood flow, right coronary artery(RCA) smooth patency, no plaque grade stenosis, TIMI3 blood flow (Figure 2). Combined with the patient's history of Cryptopteran bites, the increase of procalcitonin and high-sensitivity C-reactive protein suggested that the patient may have an inflammatory reaction. We considered that coronary spasm was caused by allergic reaction induced by the secretion of Cryptopteran.After intravenous drip of calcium gluconate injection, topical halometasone ointment, and oral loratadine treatment, the patient's symptoms improved, and the reexamination of electrocardiogram and troponin were normal (Figure 1C). The patient was followed up for 1 week after discharge, and there was no discomfort such as chest tightness and chest pain.

## Discussion

The pathogenesis of Kounis syndrome has not been fully elucidated. Some scholars believe that it is caused by inflammatory mediators such as histamine, platelet activating factor, arachidonic acid products, neutral protease, and a variety of cytokines and chemokines released in the process of allergic activation (3). The most common triggers were antibiotics (27.4%) and insect bites (23.4%) (5). Cryptopteran is a kind of beetle. When the venom comes into contact with the skin, it often causes skin inflammation, which can be manifested as blisters and rashes (6). There have been reports of nerve injury caused by toxins in China, but the author has not reported Kounis syndrome. Therefore, Kounis syndrome should be suspected even in the absence of a history of coronary heart disease when patients with a history of bites from these toxic insects present with chest pain, chest tightness, and allergic reactions (7). For type I Kounis syndrome, some scholars have suggested that in addition to mast cell activation, coronary muscle bridge is also the trigger factor, especially the anterior descending muscle bridge. Systolic compression of the muscle bridge can lead to mast cell aggregation and cause coronary spasm. This theory is also consistent with the coronary angiography results of this case (8, 9).

Management of Kounis syndrome requires treatment of both cardiac and allergic symptoms. In patients with the type I variant, treatment of allergy alone eliminates symptoms. For type II and III kounis syndrome, antiallergic therapy is as important as revascularization (10). Vasodilators such as calcium channel blockers and nitrates can be used to eliminate anaphylactogenic vasospasm (11). However, nitrates cause hypotension and impair coronary blood flow, so vasodilators are administered slowly as blood pressure permits. Screening for allergens, including an assessment of allergies to other foods, drugs, and other environmental factors, may help identify the culprit (12). Considering that intra-coronary imaging examination will increase the treatment cost of patients, and coronary angiography examination did not show diastolic intra-coronary stenosis. Therefore, intra-coronary imaging was not further performed after coronary angiography, which is indeed the limitation of this case.At present, the clinical reports of Kounis have gradually increased, and we need to accumulate clinical data and conduct experimental exploration to improve the level of diagnosis and treatment.

## Conclusion

Kunis syndrome is a life-threatening condition that can also cause myocardial ischemic injury in patients with or without coronary artery disease. Timely identification and anti-allergic treatment can achieve a good prognosis.

## Data Availability

The original contributions presented in the study are included in the article/Supplementary Material, further inquiries can be directed to the corresponding author.
